# Early detection of intravascular large B-cell lymphoma by ^18^FDG-PET/CT with diffuse FDG uptake in the lung without respiratory symptoms or chest CT abnormalities

**Published:** 2014

**Authors:** Masato Shiiba, Koji Izutsu, Makiko Ishihara

**Affiliations:** 1 Department of Diagnostic Imaging Center, Toranomon Hospital, Tokyo, Japan; 2 Department of Hematology, Toranomon Hospital, Tokyo, Japan

**Keywords:** IVLBCL, Intravascular large B-cell lymphoma, FDG-PET, Diffuse lung uptake

## Abstract

Intravascular large B-cell lymphoma (IVLBCL) is a rare and aggressive subtype of systemic extranodal non-Hodgkin diffuse large B-cell lymphoma (DLBCL). We report a rare case of IVLBCL who showed diffuse 18F-fluorodeoxyglucose (FDG) uptake in the lung in FDG-positron emission tomography/computed tomography (PET/CT) without respiratory symptoms or chest CT abnormalities. Serum biochemical studies showed a raised level of lactate dehydrogenase (LDH) and serum soluble interleukin-2 receptor (sIL-2R), which suggested the presence of malignant lymphoma strongly. A non-contrast CT showed no abnormalities in the lung fields, no lymphadenopathy was found. FDG-PET/CT revealed diffuse FDG uptake in the both lungs and in spleen as well as multiple hot spots in the liver. Under the suspicion of IVLBCL especially by the diffuse FDG uptake in the lung, a random skin biopsy was performed from three regions, the left forearm, right abdomen and left thigh in which there had been no evidence of FDG uptake. The definite diagnosis of IVLBCL was made based on the pathological analysis of the specimen from the left thigh. She achieved complete remission (CR) after combined chemoimmunotherapy. FDG-PET/CT was useful for the early detection of IVLBCL even without respiratory symptoms or any abnormal findings by chest CT.

## Introduction

Intravascular large B-cell lymphoma (IVLBCL) is a rare subtype of systemic extranodal non-Hodgkin diffuse large B-cell lymphoma (DLBCL), occurring less than 1% of all lymphomas (1). IVLBCL is characterized by the proliferation of tumor cells restricted to the lumina of vessels, capillaries in particular as defined by World Health Organization published in 2008 whereas lymph nodes are typically spared (1).

This disease is aggressive and the clinical course is deleterious when the diagnosis and treatment are delayed although the definite diagnosis is difficult to make, often made antemortem period and autopsy (1). This is due in a large part to a variety of common symptoms resulting from occlusion of small vessels by tumor cells in various tissues. Because appropriate chemotherapy in the early stage of this disease is potentially effective, comparable to conventional DLBCLs (2), the early diagnosis is important.

^18^F-fluorodeoxyglucose positron emission tomography (FDG-PET) has emerged as a powerful functional imaging tool in the assessment of non-Hodgkin lymphoma (3). However there is paucity of reports on FDG-PET in IVLBCL.

We present here a case of IVLBCL who had no respiratory symptoms which could occur in the near future nor chest computed tomography (CT) abnormalities. Diffuse FDG uptake in the lung on FDG-PET/CT was helpful in the early diagnosis of IVLBCL.

## Case Report

A 53-year-old woman was suspected a hematologic disease in a medical checking. She presented with nocturnal sweating 1 week before consulting. Her past and family history was unremarkable. Clinical examination was also unremarkable, lymphadenopathy was absent. Her hematological profile was as follows: hemoglobin: 10.4 g/dl, hematocrit: 30.9%, white blood cell count: 3.0 × 10^3^ per μl (neutrophils 57%, lymphocytes 26%, monocytes 9.5%, eosinophils 0.0% and basophils 0.0%), platelet count: 10.0 × 10^4^ per μl. Serum biochemical studies showed a raised level of lactate dehydrogenase (LDH): 849 U/L (normal range: 119-229 U/L) and serum soluble interleukin-2 receptor (sIL-2R): 2380 U/ml (145-519 U/ml), which suggested the presence of malignant lymphoma strongly.

**Figure 1:**
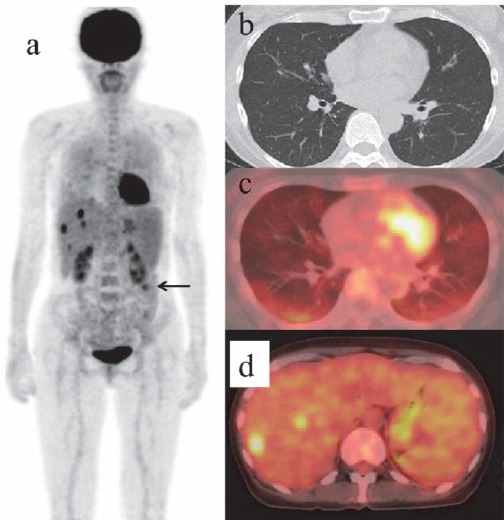
FDG-PET/CT (a, c, d) showed diffuse FDG uptake in the lung fields and in spleen and multiple hot spots in the liver. No abnormalities were detected in chest CT (b). The focal uptake inferior to the left kidney is physiological activity in small intestine (black arrow)

**Figure 2:**
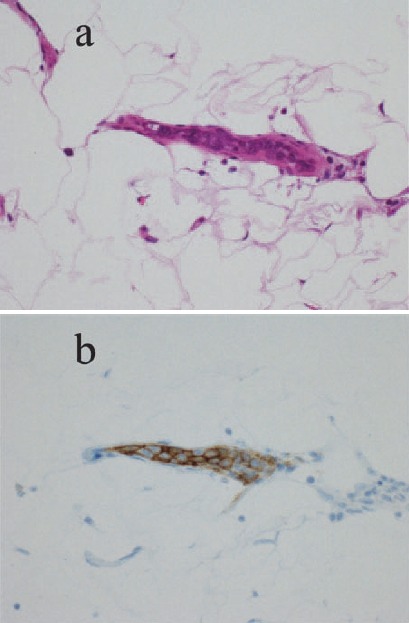
Histopathological analysis of skin biopsy from the left thigh. (a) Large lymphoma cells in the small vessels (hematoxylin and eosin staning). (b) Tumor cells were positive for CD20 (CD20 immunostain)

A non-contrast enhanced CT of chest, abdomen and pelvis was performed, some low density areas in the liver and splenomegaly were detected and no lymphadenopathy was found. No abnormalities were detected in the lung fields, although high-resolution CT was not applied. FDG-PET/CT was undertaken within two weeks after CT, revealed diffuse FDG uptake in the both lungs and in spleen as well as multiple hot spots in the liver (Figure 1). She had fever up to 38°C the next day. Three days after PET/CT a random skin biopsy was performed from three regions, the left forearm, right abdomen and left thigh in which there had been no evidence of FDG uptake. The pathological analysis of the specimen from the left thigh showed the proliferation of large lymphoma cells in the lumina of small vessels and the tumor cells were positive for CD20, demonstrating origin from B-cell lineage (Figure 2). The definite diagnosis of IVLBCL was made based on the pathological result.

**Figure 3:**
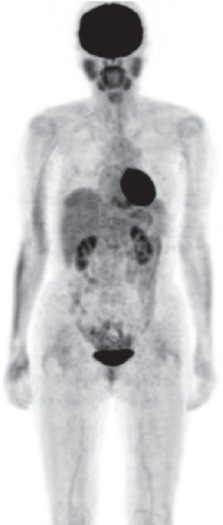
Maximum intensity projection (MIP) image of FDG-PET after the completion of combined chemoimmuno-therapy. Abnormal FDG uptakes completely disappeared

An R-CHOP chemotherapy regimen (Rituximab, cyclophosphamide, doxorubicin, vincristine and prednisolone) was started immediately. She received two courses of high dose-methotrexate (HD-HTX) and Rituximab as prophylaxis for central nervous system relapse after total of six cycles of R-CHOP. FDG-PET/CT after these therapies demonstrated complete disappearance of all abnormal uptakes including the diffuse uptake in the lungs. She achieved complete remission (CR) (Figure 3).

## Discussion

We reported a case of IVLBCL in whom abnormal FDG uptake was shown in the lung, spleen and liver. The diffuse lung uptake of FDG was especially suggestive of ILVBCL and she could be sent to random skin biopsy for definite diagnosis and prompt immunochemotherapy before emerging of respiratory or systemic symptoms.

To the extent of our search 15 case reports have been reported to date (4-18). Miura Y et al reported four patients with IVLBCL and another article reported four patients (19). In total, FDG-PET findings has been reported in 23 patients with IVLBCL and 11 cases (11/23, 47.8%) demonstrated diffuse FDG uptake in the lung (4-10). Among them, 6 cases were evaluated with chest CT too. Five of these patients (5/6, 83.3%) showed no apparent abnormalities in the lung fields similar to our case report. The other reported abnormal foci of FDG uptake were in the bone marrow (13/23, 57.0%), spleen (7/23, 30.0%), renal cortex (5/23, 22.0%), uterus/vagina (4/23, 17.4%), adrenals (3/23, 13.0%), lymph nodes (2/23, 8.7%) and stomach (1/23, 4.0%). This case report showed diffuse FDG uptake in the spleen and multiple FDG-avid lesions in the liver. Foci of FDG uptake in the reticuloendothelial systems may lead us to the possibility of IVLBCL.

In this case report no abnormal FDG uptake was detected in the cutaneous lesions, but it is reasonable to apply a random skin biopsy for the definite diagnosis of IVLBCL suggested by FDG-PET. The usefulness of random skin biopsy from unaffected skin for the definite diagnosis of IVLBCL has been proposed (20). Furthermore, Shimada et al reported that FDG-PET was able to detect only two of seven pathologically confirmed lesions (19). This suggests that the density of tumor cells might be lower than the detectability of PET imaging, but malignant cells could spread various organs without apparent signs of involvement.

In conclusion we reported a case of IVLBCL with diffuse FDG uptake in the lung without respiratory symptoms or chest CT abnormalities, which could be diagnosed early by a random skin biopsy and achieved CR with combined chemoimmunotherapy. FDG-PET provided an important information for recalling IVLBCL and could indicate random skin biopsy which could diagnose early, and lead to prompt chemotherapy, contributing to CR and long-term survival.
